# Indents

**Published:** 1907-11-15

**Authors:** 


### INDENTS.

☞ **Brief Articles of Technical or Scientific Value will receive notice and comment. Contributions invited**

**A Haemostatic.** Potassium permanganate is a valuable haemostatic used in the form of a paste made by mixing it with 4 percent. of vaselin. Dry thoroughly the part to which is to be applied. Keep in an airtight receptacle when not in use.—*Brief*.

**Putrescent Pulp.** On opening up a pulp chamber in which there is a putrescent pulp giving out a most offensive odor, dip your broach in oil of turpentine and insert in canal; the odor will change almost instantly, most agreeably to both yourself and patient. J. E. Mc Donald, in *Dominion Journal*.

**Root Canal Filling.** After pulp removal under pressure anaesthesia I always leave a small quantity of mummifying paste (zinc oxid, alum and thymol) at the extreme end of the canal and then fill with gutta-percha points dipped in a saturated solution of thymol in oil of cinnamon. The cinnamon gradually evaporates, leaving a layer of thymol crystals, lining the root canal.—Wilfred E. Griffin, *British Dental Journal*.

**Pericementitis.** Instead of using equal parts of aconite iodine and chloroform, use this prescription:

| | | |
|---|---|---|
| Rx. | Tinc. Aconiti (rad) | fluid ouncesj |
| | Chloroformi | fluid ounces iv |
| | Menthol | gr. xx. |

and you will get excellent results.—J. P. Buckley, *Dental Digest*.

